# Important Factors Affecting User Experience Design and Satisfaction of a Mobile Health App—A Case Study of Daily Yoga App

**DOI:** 10.3390/ijerph17196967

**Published:** 2020-09-23

**Authors:** Na Yu, Yi-Ting Huang

**Affiliations:** 1 Ph. D. Program in Design, Chung Yuan Christian University, Taoyuan 32023, Taiwan; yuna@qztc.edu.cn; 2 Fine Art and Design College, Quanzhou Normal University, Quanzhou 362000, China; 3 Department of Commercial Design, Chung Yuan Christian University, Taoyuan 32023, Taiwan

**Keywords:** mHealth, user experience, satisfaction, Daily Yoga, Delphi, DEMATEL, ANP

## Abstract

In recent years, mobile health (mHealth) has gained popularity. Yoga apps help users to exercise at home and improve their health. It is worth discussing how to give yogis a better experience and higher satisfaction to improve their willingness to keep using yoga apps. In this study, the Daily Yoga app was selected as the research object to explore important factors related to its user experience design and user satisfaction. Through a literature review and Delphi method composed of eight experts, this study put forward the important criteria framework of user experience design for the Daily Yoga app and then, used the DEMATEL (Decision Making and Trial Evaluation Laboratory)-based ANP (Analytic Network Process) method to determine the factors’ importance order and the causal relationships among them. Finally, combined with the results of an importance–performance analysis of 16 real users, we discuss the improvement measures. The research results show that the yoga class is the most critical factor in the user experience design of the Daily Yoga app, the target plan is a factor that is in great need of improvement, and having an attractive interface can improve user experience. The evaluation model of the study can act as a reference for improving user experience with the Daily Yoga app, and can also be widely used in the process of user experience design, questionnaire production, and evaluation optimization of mHealth app and related applications.

## 1. Introduction

Yoga is an ancient mind–body practice that originated in India and can be traced back to 3000 BC. Traditionally, yoga is a comprehensive experience that integrates body posture, breathing exercises, internal awareness, concentration, and meditation [1]. Nowadays, with the spread of yoga culture, yoga is not only a method for improving fitness, decompression, and body shaping, but it is increasingly becoming representative of a healthy lifestyle, which has been widely publicized. The literature has shown that yoga practice can optimize overall health and quality of life. The functions of yoga include increased muscle strength, balance, and flexibility [2]; improved sleep quality for insomniacs [3]; reduced incidence of chronic disease and delayed aging [4,5]; reduced heart rate and blood pressure; assistance in weight loss [6]; and reduced psychological problems such as stress, anxiety, and depression [7].

Mobile health (mHealth) involves the use of mobile devices (such as mobile phones and personal digital assistants) to provide users with health information and medical services [8]. Mobile device technology supports and promotes health information technology (HIT), which aims to improve global health [9]. Based on the statistics related to mHealth apps usage, mobile apps are effective for improving physical activity and healthy eating habits [10]. Mobile devices have many advantages, such as providing users with real-time information, reminding them of their daily work (physical exercise, diet, etc.), providing guidance and suggestions, and enhancing their determination to make changes when faced with challenges [11]. Studies have shown that health interventions in mobile applications are common and effective ways to improve health behaviors in the general population [12]. With the development of the mobile internet industry and the popularization of smart mobile devices, health-themed mobile apps have proliferated [13]. The technological revolution in the field of health has led to the development of better and more sustainable services for people.

In the past decade, the use of mobile devices in China has rapidly become popular, and mHealth apps are gaining popularity [14]. China’s health system is facing huge and continuous challenges, and the unique features of mHealth provide opportunities to overcome these challenges [15]. Labrique et al. introduced the general categories and descriptions of mHealth in detail [16]. According to the literature, yoga apps also belong to the category of mHealth. In China, it is common for self-practicing yogis to accept online video teaching. Among those who follow yoga videos, 60% of them choose yoga teaching apps [17]. Due to the outbreak and spread of COVID-19, exercise at home is becoming a new way of life for the general public [18]. It has become a trend to achieve a healthy lifestyle through mobile device training. In China, yoga apps include Daily Yoga, Wake Yoga, Yoga Easy, Yoga TV, Omma Yoga, Yoga Wave, Yoga Lemon, All Yoga, and Lotus Yoga [19]. Founded in 2012, Daily Yoga ranks no.1 in both the global and Chinese yoga app markets, with more than 50 million users in 212 countries, and China has 20 million users in more than 400 cities [17]. Online yoga apps not only provide convenience for yoga enthusiasts and bring users a different experience from the traditional yoga studio, but also better spread the yoga culture through internet technology.

According to the definition of ISO 9241-210, user experience is a cognitive impression and response to a product, system, or service used or expected to be used [20]. The definition is supplemented with the following explanation: user experience refers to all the feelings of users before, during, and after using a product or system, including emotions, beliefs, preferences, cognitive impressions, physical and psychological reactions, behaviors, achievements, etc. [20]. User experience is the result of a system, product, or service’s brand image, manifestation, function, system performance, interactive behavior, and auxiliary capability [20]. The user experience also stems from the user’s internal and physical state, which comes from the user’s previous experience, attitude, skill, ability, and personality, as well as the use environment [20]. User satisfaction refers to the feeling of a state of pleasure or disappointment formed by comparing the perceived effect of a product with his/her expected value [21]. The results of service quality and interaction quality significantly affect customer experience quality, thus affecting customer loyalty [22]. An easily identifiable interface and effective tutorials improve user satisfaction [23]. User experience can also enhance the market competitiveness of products and increase the revenue gained by enterprises [24]. Decisions affect the optimization direction of the app, and the future of the enterprise. Due to the constraints of the enterprise’s operating budget, it is necessary to determine the priority of improvement factors. The DANP (DEMATEL (Decision Making and Trial Evaluation Laboratory)-based ANP (Analytic Network Process)) combines DEMATEL and ANP and lets the two kinds of information jointly determine the key factors [25].

As mentioned above, achievement of a healthy lifestyle through yoga practice can prevent or reduce many disease and health risks. User experience design has a great impact on user satisfaction and continuous use intention of the app. Greater mHealth app usage is effective for improving health [10]. At present, most of the articles on yoga and mHealth focus on their effects on physical and psychological improvement; however, few articles are related to the user experience of yoga apps. From the above discussion, it is clear that it is important to evaluate the user experience design of yoga apps and improve the strategy related to user satisfaction. The unsolved questions include the following: What are the main and key factors that affect the user experience of yoga apps? What is the relationship between the user experience factors of yoga apps? What is the difference between the user experience evaluation criteria of yoga apps and general apps? Compared with other mHealth, what is the innovation in the user experience evaluation criteria of yoga apps proposed in this study?

In this study, the Daily Yoga app (Daily Yoga Culture Technology Co., Ltd., Xi’an, China), which has the largest number of users and is the most representative app, was selected as the research object, and the user experience design and satisfaction of the Daily Yoga app were taken as the themes. Through a literature review and case analysis methods to screen the preliminary criteria, the Delphi expert method was used to build a formal framework of the criteria. The DANP method was used to analyze the criteria and draw a causal diagram, and finally, an empirical importance–performance analysis (IPA) was done. This study aimed to (1) propose important evaluation criteria that affect the user experience design of the Daily Yoga app through the Delphi method to improve user experience satisfaction; (2) evaluate key factors more accurately by using the combination of DEMATEL and ANP and draw a network relationship map; and (3) conduct an importance–performance analysis through a satisfaction questionnaire. This paper is divided into six parts: the first part is the introduction, which introduces the research motivation, purpose, methodology, and results of this paper; the second part is a literature review on yoga, user experience, experiential marketing, and DANP; the third, fourth, and fifth parts introduce the methods, results, and discussion respectively; and the last part presents the conclusions.

## 2. Theoretical Background

### 2.1. Yoga App and User Experience

The main motivation for the Chinese population to practice yoga is to improve their physical and mental states [26]. When choosing a yoga brand, users focus on course design, particularly the course content. They think that the quality of the course is very important [17]. Telles et al. studied the motivation and choices of yoga participants and found that the most common reason for respondents to practice yoga was to improve their physical health, and people of different ages had significant differences in their choices of yoga practice items [27]. Gard et al. divided yoga into four categories for self-regulation, including moral code, maintaining posture, breathing regulation, and meditation techniques, and constructed a model to promote the self-regulation of mental and physical health through yoga [28]. In a study by Uebelacker et al., yoga practitioners completed daily and weekly online yoga practice assessments within 28 days, and the results indicated that it was reasonable to use weekly practice assessment tools to evaluate the number of courses attended [29].

Yoga apps belong to the mHealth category. However, there are many specific application categories in mHealth, and the existing literature related to user experience design is rarely directed at yoga apps. This study focused on the user experience design factors of mHealth and related applications. MHealth apps include those that provide educational resources, self-monitoring tools, social support, interactive training modules, and incentive tools [30]. Martínez-Pérez et al. developed a framework to evaluate the quality of mHealth experience, including content quality, performance, ease of use, availability, learning, security, and precision [31]. Common benefits of mHealth weight loss apps include self-monitoring, goal setting, feedback, motivation, education, and reminders, and the key factors are personalization, attractiveness, and engagement [32]. Key features of successful mHealth interventions include self-monitoring, feedback, social support, personal customization, education, prompting, reminding, and motivating participants [33,34]. Feedback and rewards are the most common incentives used in the gamification of e-health apps, and socialization has attracted a lot of attention in gamified apps [35]. MHealth consumers have behavioral intentions related to the measurement, storage, and management of health data [36]. Enterprises need to understand the intentions and behaviors of healthy consumers in order to develop and implement effective and efficient strategies. Users of mental health apps pay more attention to the friendliness of the user interface and like the apps to provide them with various options, functions, and content [37].

Attitudes, subjective norms, and positive and negative expectations have significant impacts on consumers’ willingness to buy sporting goods online, and past behavior and frequency of desire affect consumers’ intentions [38]. Pang et al. showed that perceived usefulness and satisfaction have important impacts on the continuous intention to use knowledge sharing platforms, which should strengthen their content quality control to ensure consistency between service quality and payment, so as to improve platform quality [39]. Customer participation in brand building activities has direct and indirect impacts on satisfaction and loyalty, which are partly influenced by brand experience. Therefore, companies should consider how to stimulate brand experience through co-construction [40]. Hassenzahl and Tractinsky mentioned that user experience includes three factors: emotion, the experiential, and beyond the instrumental [41]. Users experience augmented reality by following the mascot’s instructions through the mobile device with the app installed, thus improving the satisfaction of the whole experience [42]. Having a sense of control over the user interface will directly affect use efficiency, and customizability is positively proportional to the user’s perceived ease of use, which can improve the user’s satisfaction [43]. The key to a better user experience and greater loyalty are a sense of accomplishment, self-fulfillment, belonging, fun, convenience, and better service [44]. Laugwitz et al. established an evaluation questionnaire regarding user experience, including attractiveness, dependability, stimulation, perspicuity, efficiency, and novelty [45]. According to Finstad, the usability indicators of user experience include satisfaction, effectiveness, efficiency, and overall [46].

### 2.2. Strategic Experiential Modules as User Experience Evaluation Dimensions

Schmitt proposed strategic experiential modules (SEMs) with the ultimate goal being to create a holistic experience including sensory experiences (sense); affective experiences (feel); creative cognitive experiences (think); physical experiences, behaviors, and lifestyles (act); and social identity experiences (relate) [47]. SEMs are closely related to the content of the user experience described above. In recent years, some scholars have applied experiential marketing theory to relevant research on user experience. Rudposhti et al. used the technology acceptance model (TAM) and experiential marketing theory to conduct a questionnaire survey on Iranian Facebook users and found that users’ perceived usefulness and user experience affect users’ attitudes towards applications, and perceived ease of use was found to have a positive impact on perceived usefulness [48]. Lo and Wang evaluated the user experience of unmanned stores from three dimensions: economic experience, marketing experience, and feeling experience [49]. Tasci and Milman used SEMs to develop a scale to measure tourists’ experience in theme parks, which was to fill in the research on the components of theme park experience [50]. Hsu et al. adopted the theory of user experience, utilitarian and hedonic attitudes, and experiential marketing, and their results showed that user experience and experience value are significantly positively correlated which, in turn, affects attitude [51].

Daily Yoga is a global yoga service brand that integrates fitness and culture. It includes an online yoga service, content output, e-commerce, offline yoga training, and yoga cultural activities [17]. This study focused on user experience with the Daily Yoga app and selected SEMs as the five dimensions of user experience design to examine. In addition, based on the discussion of the other literature and case analyses, the corresponding preliminary user experience design factors were proposed. These factors included having an attractive interface, consistency, graphics, color, sound effects, and identification in the sensory experience dimension; immersion, incentive, interest, sense of accomplishment, and human kindness in the emotional experience dimension; creative feedback, flexible choice, guided use, and resonant copywriting in the thinking dimension; yoga class, data records, professional guidance, target plan, yoga mall, and participation in the action experience dimension; and social attributes, trustworthiness, brand recognition, and activity in the related experience dimension.

### 2.3. DEMATEL-Based ANP Used to Discuss Key Factors

There are many multicriteria research methods that can be used to explore key factors. Among these, the ANP proposed by Saaty is a very effective tool that can be used to prioritize the criteria in an organized way [52]. A team of experts develops scales and allocates resources according to priorities. ANP is widely used to resolve inter-relationships between key criteria and to analyze the importance of the criteria. DEMATEL is used to solve complex and tangled problems and can improve the understanding of special problems and provide feasible solutions [53]. The most important feature of DEMATEL is that it can be used to explain the relationships among dimensions or clusters, and it can obtain the core criteria of dimensions or determine effective representative elements. Different from the simple combination of traditional DEMATEL and ANP methods, the DEMATEL-based ANP (DANP) can use the total influence matrix directly as the unweighted super matrix of the ANP, avoiding the pairwise comparison operation that is traditionally required by ANP methods [54]. The DANP method provides formal decision-making techniques to evaluate limited factors and summarizes the evaluation results to determine specific strategies. In recent years, the DANP has been widely used to assess user experience, marketing strategies, control systems, and sustainable management. Kao et al. explored the factors influencing wearable trackers with the DEMATEL method [55]. Based on the DANP method and the improved VlseKriterijumska Optimizacija I Kompromisno resenje (VIKOR) method, Liu et al. determined the key factors influencing the adoption of sustainable mobile healthcare [56].

## 3. Methods

In this paper, using the literature review method, the SEMs were selected as the five dimensions of user experience on yoga apps. In addition, Delphi, DANP, and IPA were used as quantitative research methods. The Delphi method was used to establish important criteria related to user experience design on the Daily Yoga app through expert questionnaires. The DEMATEL-based ANP method was used to determine key factors and build a Network Relationship Map (NRM). The IPA method was used to analyze the overall situation related to user experience design criteria and corresponding satisfaction on the Daily Yoga app. 

The research plan, questionnaire, and consent form of the participants mentioned in the above study design were reviewed by the Research Ethics Committee. Experts and users who participated in this study filled in the informed consent form.

### 3.1. Delphi Method

The Delphi method is a group decision making method. The items in the Modified Delphi method were proposed based on the related body of literature, and experts put forward their opinions in the first round of the semi-structured questionnaire [57]. In this study, four staff from the Daily Yoga app design team and four teachers from universities with app design experience formed an expert group (Table 1).

### 3.2. DEMATEL-Based ANP

**Figure 1:**
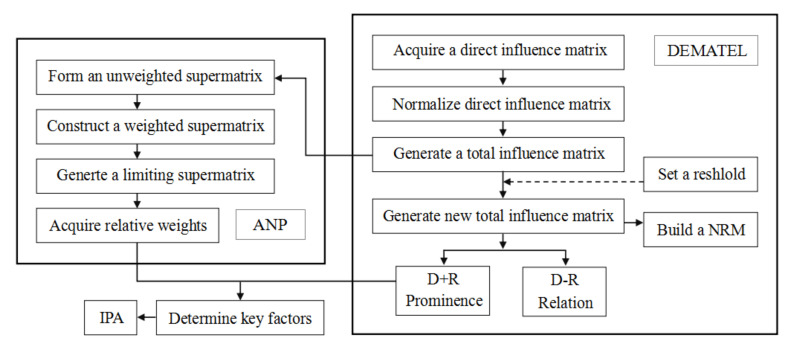
DANP framework. ANP—Analytic Network Process; DEMATEL—Decision Making and Trial Evaluation Laboratory; IPA—importance–performance analysis; NRM—Network Relationship Map.

The DANP operating architecture, as shown in Figure 1, was adopted in this study. The combination of DEMATEL and ANP can not only rank the importance of the criteria but can also determine the causal relationships among the criteria.

#### 3.2.1. Research Questionnaire

According to the 10 criteria in the formal research framework, an influence degree questionnaire was designed. As shown in Table 2, a score of 0–2 was used as the rating scale to indicate the impact of the criteria.

#### 3.2.2. Establishment of the Direct Influence Matrix Z

Experts judged the degree of influence of the criteria and filled in the value defined in step 1. After the integration of the questionnaires completed by each expert, a direct influence matrix Z was established, as shown in Equation (1), where n represents the number of indicators, a_ij_ represents the degree to which criterion i influenced criterion j, and the value of the diagonal part was set to 0.

(1) $$A=\left[\begin{array}{ccccc} a_{11} & \ldots & a_{1j} & \ldots & a_{1n}\\ \begin{array}{l} .\\ .\\ . \end{array} & . & \begin{array}{l} .\\ .\\ . \end{array} & . & \begin{array}{l} .\\ .\\ . \end{array}\\ a_{i1} & \ldots & a_{ij} & \ldots & a_{in}\\ \begin{array}{l} .\\ .\\ . \end{array} & . & \begin{array}{l} .\\ .\\ . \end{array} & . & \begin{array}{l} .\\ .\\ . \end{array}\\ a_{n1} & \ldots & a_{nj} & \ldots & a_{nn} \end{array}\right]$$

#### 3.2.3. Establishment of the Normalized Direct Influence Matrix X = S ∗ Z

The normalized direct influence matrix X was obtained by standardizing the direct influence matrix Z. Normalization methods are shown in Equations (2) and (3):

(2) $$S=\min\left[\begin{array}{ccc} \frac{1}{\max i\sum_{j=1}^{n}\left|a_{ij}\right|} & , & \frac{1}{\max j\sum_{i=1}^{n}\left|a_{ij}\right|} \end{array}\right]$$

(3) X=S×A

#### 3.2.4. Calculation of the Total Influence Matrix T

T is the total impact matrix, when $\lim_{k\to \infty }(X{)}^{k}=0$. The equation is as follows:

(4) $$T=\sum_{k=1}^{\infty }X^{k}=X{\left(I-X\right)}^{-1}.$$

T_ij_ (i, j = 1, 2, …, *n*) is an element in the total influence matrix T, and I is the identity matrix with a diagonal value of one.

#### 3.2.5. Setting a Threshold and Building an NRM

According to matrix T obtained in step 4, α was set as the threshold (α was determined by experts). If the number in the T matrix was less than α, it was replaced by 0. If the number in matrix T was greater than α, it was retained. In this step, a new total influence matrix T * was generated to build a more concise NRM. When an arrow pointed from one factor to another, it meant that the former affected the latter.

#### 3.2.6. Importance and Relation of Each Criterion

The importance (D + R) and relation (D – R) of each criterion were obtained from the new total impact matrix T *. The sum of the elements in each row was D, and this represented the degree to which a criterion directly or indirectly affected the other criteria. The sum of the elements in each column was R, which indicated the degree to which a criterion was directly or indirectly affected by other criteria. The higher the value of (D + R), the more important the criterion. If the (D – R) of the criterion was positive, this indicated that the criterion tended to be a cause. If the (D – R) of the criterion was negative, this indicated that the criterion tended to be an effect.

#### 3.2.7. Ranking the Importance of Factors

The total influence matrix of DEMATEL was taken as the unweighted super matrix in the ANP operation, and the matrix was normalized. The normalized matrix was multiplied by itself until it converged to obtain a limiting super matrix. The relative weight of each criterion was determined by the limiting super matrix. According to the sum of the DEMATEL ranking and DANP ranking, a smaller ranking sum indicated a more important factor.

### 3.3. Importance–Performance Analysis

IPA is an easy-to-use technique for measuring the importance and performance of attributes to further develop effective plans and optimize resource allocation [58]. Hinderks et al. proposed a method to evaluate the results of the user experience questionnaire using IPA and made suggestions from the results [59]. Based on the IPA method, the advantages and disadvantages of a case are analyzed, and the resources are reasonably allocated. The IPA generates four quadrants on the vertical axis of importance and the horizontal axis of performance. The quadrant with high importance and low performance represents key areas that need improvement, and the quadrant with high importance and high performance represents the continuous holding area. The quadrant with low importance and low performance represents the lower priority areas, and the quadrant with low importance and high performance represents the oversupply areas.

## 4. Results

### 4.1. Establishment of a Framework for Criteria Evaluation

Two rounds of the Delphi method were conducted in this study. In the first round, the experts scored the preliminary factor architecture and proposed corresponding modification suggestions. In the second round, experts agreed on the revised factors. Eventually, a formal research framework with 5 dimensions and 10 criteria was established. The 10 revised criteria and their descriptions are shown in Table 3.

The threshold value of consensus difference set in this study was (CDI) ≤ 0.1. In the first round of the questionnaire, the experts gave an initial score of each criterion and suggested whether the criterion should be modified, added, or deleted. Criteria with CDI > 0.1 included identification and yoga class, for which a consensus could not be reached. The second round of the questionnaire was completed on the basis of the revised first round of the questionnaire. Experts reached a consensus on the 10 selected criteria, and the CDI values of the 10 evaluation criteria were all ≤0.1. Nine criteria used in this study were based on scores from high to low. There was another criterion used, yoga mall, which did not receive a high score. However, this criterion is a unique experience module in the Daily Yoga app compared with other Chinese yoga apps. The criterion was also included in the final formal research framework.

### 4.2. Ranking the Importance of the Criteria

According to the total influence matrix T shown in Table 4, 0.369 was found to be the average value of all elements. If the value in matrix T was less than 0.369, it was replaced by 0. If the value in the T matrix was more than 0.369, it was retained. Therefore, a new total influence matrix T * was generated.

According to the new total influence matrix T *, the values of prominence (D + R) and relation (D – R) were calculated, as shown in Table 5. The results showed that the (D + R) value of C6 (yoga class) was the largest, and this was therefore considered to be the central influencing factor. The (D – R) value of C1 (beautiful interface) was the largest, and this was considered to be a major contributing factor. However, the (D – R) value of C10 (brand recognition) was the smallest, and this was most affected by other criteria and was considered to be a result factor.

The overall ranking of the criteria is shown in Table 6. In this study, based on the first half of the sequence, the key criteria were selected, and these included C6 (yoga class), C1 (attractive interface), C2 (identification), C8 (target plan), and C7 (data record), the five key factors involved in the user experience design of the Daily Yoga app.

### 4.3. Building a Network Relationship Map

**Figure 2:**
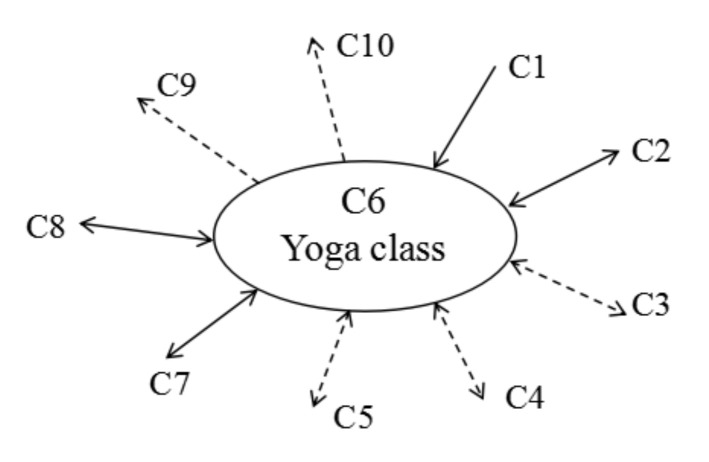
Simplified NRM.

In order to determine the important relationship within the criteria, a simplified causal diagram of the criteria was drawn based on the new total influence matrix T *. For example, a causal diagram with the most important “yoga class” factor as the core is shown in Figure 2. It can be seen from the figure that C6 (yoga class) interacts with C2 (identification), C8 (target plan), C7 (data record), C3 (sense of achievement), C4 (incentive), and C5 (guided use). In addition, C1 (attractive interface) affects C6 (yoga class). C6 (yoga class) affects C10 (brand recognition) and C9 (yoga mall). Solid arrows are used to indicate the influences of key criteria, and dotted arrows are used to indicate the influences of non-key criteria. Combined with Table 5, the higher the value of (D – R), the better its effect as an improvement. Therefore, the C1 (attractive interface) criterion was considered to be the source criterion.

### 4.4. IPA Results

In order to present the performance of the 10 criteria, eight experts and eight ordinary users were investigated in this study. Respondents used a 5-point Likert scale to rate satisfaction issues related to user experience design criteria of the Daily Yoga app. The average result of each option was calculated to obtain the satisfaction score for the Daily Yoga app user experience.

**Figure 3:**
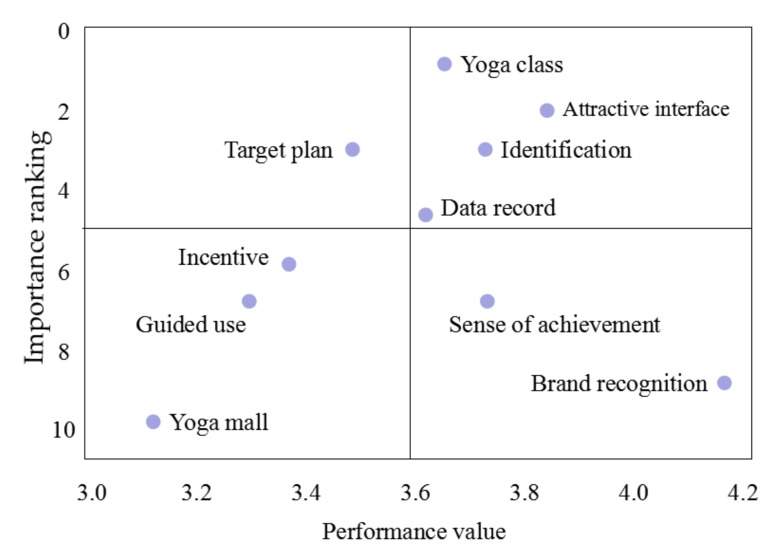
Importance–Performance Analysis (IPA) of the Daily Yoga app.

The main purpose of the IPA is to examine indicators of improvement, persistence, overemphasis, and neglect in terms of performance values and relative importance. Different from the traditional IPA, in this study, the weight part of the criterion was replaced by the overall ranking shown in Figure 3—the lower the score, the higher the importance. In the satisfaction questionnaire used in this study, some criteria had multiple related questions to allow the specific situation to be analyzed further. The satisfaction score for each criterion shown in Figure 3 refers to the overall average score for the criterion. The approximate average value of all criteria performance values was 3.6 points, which was taken as the threshold value. The importance–performance analysis is shown in Figure 3. Criteria with more than 3.6 points had a higher level of satisfaction and should be maintained. However, criteria with less than 3.6 points need to be improved.

## 5. Discussion

### 5.1. Factor Analysis

After carrying out the Delphi method, 10 important criteria were selected, which were yoga class, attractive interface, identification, target plan, data records, incentive, guided use, sense of achievement, brand recognition, and yoga mall. On this basis, the importance ranking and performance value of each criterion were further obtained through the DANP and IPA methods. In order to comprehensively analyze the factors and satisfaction of user experience design on the Daily Yoga app, it is necessary to discuss the 10 criteria used.

#### 5.1.1. Key Factors with High Satisfaction

The course content of the Daily Yoga app is relatively comprehensive and rich, with clear and simple teaching pictures and detailed step-by-step guidance. The yoga class is a key criterion in the user experience design of the Daily Yoga app. However, when asked about more available paid yoga classes, users’ scores for the corresponding options were significantly lower—only 3.56 points. Determining how to guide users to buy yoga classes voluntarily can be inspired by the above research results, that is, determining how mHealth apps can guide free users to buy the full version of the application in the future. Yoga classes are strongly influenced by having an attractive interface, identification, data records, and target plan, but they are less influenced by encouraging a sense of achievement, incentive, and guided use. The results show that the aesthetic and personalized interface presentation, combined with having a professional and attractive target plan in the data recording module, as well as efficient guidance and incentives, can stimulate users’ willingness to pay for courses.

The importance and satisfaction of having an attractive interface and identification of the Daily Yoga app are relatively high. According to the satisfaction questionnaire results, users’ satisfaction with the color of the Daily Yoga app was as high, 4.13 points, and the score for agreeing with the use of the cute and amiable mascot was 3.81 points. Other options related to aesthetics and recognition (such as attractiveness and personalization) scored slightly lower, but all were above the threshold of 3.6 points. Having an attractive interface affects all of the other criteria, identification affects most of the other criteria, and these two criteria are important contributing factors. Having an attractive interface and identification influence each other, and these are key factors in the user experience design of the Daily Yoga app. Therefore, it is necessary to maintain the advantages of these two criteria to improve overall user experience satisfaction.

The data record helps users to record exercise expenditure and manage and evaluate physical fitness. This criterion is a causal factor, which has relatively strong influences on the sense of achievement, yoga class, and target plan and relatively weak influences on incentive, brand recognition, and yoga mall. At the same time, the data record is influenced by the factors yoga class, target plan, and attractive interface. The data record allows users to fully understand their practice, which encourages them practice and improves their sense of accomplishment. The data record is a key factor in the user experience design of the Daily Yoga app and has a satisfaction score of 3.63 points, slightly above the threshold of 3.6 points. The advantages of the factor should be appropriately maintained, but there is still room for improvement. Health diaries are considered easy to use and useful for health management [60]. It is suggested that the Daily Yoga app could provide a module that allows users to fill in a health diary while presenting data records.

#### 5.1.2. Key Factors Associated with Low Satisfaction

According to the results of empirical research, the target plan is a key criterion, but its performance is poor, so it is in urgent need of improvement. The target plan criterion interacts with the factors yoga class, data record, and incentive in a highly correlated manner. At the same time, the target plan criterion is affected by guided use, having an attractive interface, and identification. In addition, the target plan affects the sense of accomplishment. When designing an application, it is still important to make sure that it is appealing to the users, and content should be tailored to different target groups [32]. At present, the target guidance of the Daily Yoga app is only displayed when users log in for the first time, and users cannot make a comprehensive target plan conveniently in the later stage. The Daily Yoga app has a large number of users. Due to the wide distribution of users’ ages, occupations, and characteristics, it is necessary to provide more reasonable, appropriate, and clear fitness goals and plans.

#### 5.1.3. Factors of General Importance and Low Satisfaction

Incentive, guided use, and yoga mall are factors that have general importance and low satisfaction in user experience design, which should be improved when the target planning criterion is given priority. The encouragement, supervision, and reward mechanism of the Daily Yoga app is 3.38 points, which is relatively low. This may be due to the fact that more than half of the yoga users in China are driven by internal forces to practice yoga, rather than being influenced by the outside world [17]. This does not mean that the user experience design of this criterion is not good. Guided use has an impact on yoga classes and so on, but the current satisfaction score is only 3.31 points, so it needs to be improved. Yoga mall is a strong outcome factor with low importance and user satisfaction. When the overall user experience and satisfaction of the Daily Yoga app is improved, the yoga mall may attract users. Under the condition of abundant enterprise resources, we can start to improve the user experience design of the factor yoga mall.

#### 5.1.4. Factors of General Importance and High Satisfaction

Sense of achievement and brand recognition are two criteria with general importance. Both of them are resulting factors, and they are influenced by most other criteria. In terms of satisfaction, brand identity scored high with 4.19 points, while sense of accomplishment scored 3.75 points. This shows that users agree with Daily Yoga’s guidance and culture and feel that the services are trustworthy. Users generally feel that their physical fitness, yoga skills, and knowledge level have improved after using the Daily Yoga app. Therefore, there is no need to pay too much attention to sense of achievement and brand identity.

### 5.2. Implications for Research

Currently, most articles on yoga and mHealth focus on the effects on physical and mental improvement. From the perspective of user experience, this study proposed the important influencing factor framework of yoga mHealth apps to fill the gaps in the research field. The framework established in this study was different from the general user experience-related literature proposed by other scholars, such as user experience usability indicators [46] and user experience questionnaire construction [45]. Instead, this study focused more on mHealth apps. However, based on the latest user experience definition and the content of SEMs, the proposed user experience criteria were not exactly the same as other scholars’ literature on mHealth user experience.

The criteria framework established in this study can be used in the process of user experience design, questionnaire production, evaluation, and optimization of mHealth and related applications. In addition, the newly proposed criteria are innovative and valuable for research. In this study, the DANP method was adopted to find the strong or weak influence relationship among factors. This allows decision makers to analyze factors in a global and interactive way instead of a single perspective. This study was an empirical study, and the research results provide decision-making advice for daily yoga enterprise to optimize the application, which has practical value.

Among the 10 criteria proposed in this study, the factors of yoga class and yoga mall make yoga apps different from other apps, and are also the criteria that did not appear in the relevant literature. Yoga class was the most important core factor, while yoga mall was the least important. Both can be changed according to specific mHealth themes, such as dance class and dance mall. Target plan and data recording are common factors for mHealth apps, yet they are rarely mentioned in the existing user experience research framework for mHealth apps. The criteria of incentive and achievement are not only suitable for mHealth apps, but also for games and task completion apps.

Guided use is a relatively universal criterion for all types of apps. Attractive interface and recognition are closely related. Attractive interface almost always presents in other research frameworks; however, the criterion of recognition proposed in this paper is also important, as a differentiated product is more likely to win in a market where many similar products compete fiercely. In addition, the criterion of brand recognition extracted from SEMs theory conforms to the definition of user experience in recent years, and good brand recognition is conducive to the continuous use of products by users.

## 6. Conclusions

The rapid development of mobile device technology and HIT has spawned a large number of mHealth apps in a short period of time. This literature review indicates that the evaluation framework for the user experience design of yoga apps has not been established so far. Using Schmitt’s SEMs, the user experience design criteria of the Daily Yoga app were divided into five dimensions: sense, feel, think, act, and relate. After a literature review and two rounds of the Delphi method, there were 10 relatively important criteria to pave the way for subsequent research. The DANP method was used to obtain the importance ranking and NRM of 10 criteria. In order of importance, from high to low, the criteria ranking is yoga class, beautiful interface, juxtaposition of identification and target plan, data record, incentive, juxtaposition of guided use and sense of achievement, brand recognition, and yoga mall. Using the DANP method, the key factors related to user experience design on the Daily Yoga app can be effectively evaluated. The IPA diagram of the Daily Yoga app shows that the yoga class, having an attractive interface, identification, and data record are key factors with high satisfaction. The advantages of these criteria can be maintained, but there is still room for improvement. Target plan is a key criterion with low satisfaction, and we urgently need to further improve the user experience design of this criterion. Incentive, guided use, and yoga mall are factors with general importance and low satisfaction, which can be improved in cases with abundant enterprise resources. Sense of achievement and brand recognition are factors with general importance and high satisfaction, which do not require too much attention.

Based on academic theoretical knowledge and real cases, this study combined the importance of criteria and user satisfaction to analyze a specific situation. We have provided enterprises with an evaluation framework for user experience design on the Daily Yoga app, and we have provided reference suggestions for improving the user experience satisfaction of the product so as to further enhance users’ intentions to use mHealth applications and promote their physical and mental health. We provide a model of evaluation framework through this study, and this preliminary contribution can be provided to other authors in the same field for further test verification and improvement. The management implication of user experience design proposed in this paper may only apply to the Chinese version of the Daily Yoga app, but it may not be suitable for the international version of the Daily Yoga app. Therefore, in follow-up research, efforts should be made to collect data about user experience design and satisfaction with the international version of the Daily Yoga app to improve the international applicability of this study. In addition, this study can provide referable research ideas for similar mHealth apps, but it may not be completely applicable to the user experience design and satisfaction improvement of other yoga apps.

## Figures and Tables

**Table 1:** Backgrounds of Delphi respondents.

No.	Job	Experience
1	Product manager	Product Manager of the Daily Yoga app with 7 years working experience as the app product manager.
2	Product manager	Product Manager of the Daily Yoga app with 5 years working experience as the app product manager.
3	User interface designer	UI designer for the Daily Yoga app with 5 years of app interface design and visual design experience.
4	User interface designer	UI designer for the Daily Yoga app with 5 years of app interface design and visual design experience.
5	University teachers	18 years of work experience, PhD in design, assistant professor, creative director. Research fields: web design, graphic design, packaging design, identification design, and brand design.
6	University teachers	7 years of teaching experience in a university, PhD in design. Research fields: visual communication design, UI design direction.
7	University teachers	5 years of teaching experience in a university, PhD in design. Research fields: industrial design, interaction design direction.
8	University teachers	5 years of teaching experience in a university, PhD in design, design director. Research fields: visual communication design, UI design, brand design.

**Table 2:** Rating scale.

Criteria	0	1	2
Association	No effect	Some effect	Strong effect

**Table 3:** Descriptions of the revised assessment criteria.

Dimensions	Criteria	Criteria Descriptions
Sense	Attractive interface (C1)	The visual design of the interface is exquisite, the overall appearance is consistent, and the color collocation is visually pleasing.
	Identification (C2)	The interface design is featured, personalized and identifiable, making it easy for people to remember the Daily Yoga app.
Feel	Sense of achievement (C3)	The participants’ physical fitness, skills, and knowledge of yoga have steadily improved.
	Incentive (C4)	Encouragement, supervision, and reward mechanisms have the effect of cultivating regular yoga practice.
Think	Guided use (C5)	Provides simple and clear guidance and instructions for users to use the app more effectively.
Act	Yoga class (C6)	Some quality yoga classes are available for free, and more classes are available if you pay for them.
	Data record (C7)	It helps users to record exercise expenditure and manage and evaluate physical fitness.
	Target plan (C8)	Provides a plan to achieve goals with clear steps for fitness tasks.
	Yoga mall (C9)	Meets the needs of users to purchase peripheral yoga equipment more quickly and conveniently.
Relate	Brand recognition (C10)	Is in agreement with the guidance and culture of Daily Yoga and believes that its service quality is trustworthy.

**Table 4:** Total influence matrix T.

T	C1	C2	C3	C4	C5	C6	C7	C8	C9	C10
C1	0.287	0.426	0.442	0.474	0.455	0.547	0.387	0.467	0.491	0.541
C2	0.388	0.280	0.413	0.423	0.407	0.485	0.363	0.415	0.441	0.488
C3	0.229	0.253	0.283	0.366	0.317	0.380	0.308	0.341	0.336	0.392
C4	0.265	0.289	0.415	0.302	0.325	0.404	0.359	0.421	0.345	0.393
C5	0.280	0.330	0.396	0.369	0.270	0.440	0.333	0.369	0.349	0.398
C6	0.337	0.375	0.527	0.497	0.435	0.420	0.459	0.497	0.462	0.519
C7	0.285	0.311	0.461	0.433	0.367	0.452	0.291	0.450	0.398	0.407
C8	0.272	0.273	0.447	0.426	0.345	0.459	0.372	0.317	0.365	0.390
C9	0.242	0.246	0.272	0.283	0.269	0.325	0.255	0.293	0.228	0.355
C10	0.275	0.289	0.351	0.327	0.300	0.362	0.293	0.336	0.346	0.290

**Table 5:** Prominence and relation of each criterion.

No.	Criteria	D	R	D + R	D − R
C1	Attractive interface	4.230	0.388	4.618	3.842
C2	Identification	3.460	0.801	4.261	2.659
C3	Sense of achievement	0.772	3.102	3.874	−2.330
C4	Incentive	1.633	2.622	4.255	−0.989
C5	Guided use	1.971	1.298	3.269	0.673
C6	Yoga class	4.190	3.586	7.776	0.604
C7	Data record	2.600	1.217	3.817	1.383
C8	Target plan	2.094	2.619	4.713	−0.525
C9	Yoga mall	0.000	1.792	1.792	−1.792
C10	Brand recognition	0.000	3.527	3.527	−3.527

**Table 6:** The overall ranking of the criteria.

No.	Criteria	DEMATEL	DANP	Sum of Ranks	Overall Ranking
C1	Attractive interface	3	2	5	2
C2	Identification	4	3	7	3
C3	Sense of achievement	6	9	15	7
C4	Incentive	5	7	12	6
C5	Guided use	9	6	15	7
C6	Yoga class	1	1	2	1
C7	Data record	7	4	11	5
C8	Target plan	2	5	7	3
C9	Yoga mall	10	10	20	10
C10	Brand recognition	8	8	16	9
